# Integrated metabolomic and transcriptomic dynamic profiles of endopleura coloration during fruit maturation in three walnut cultivars

**DOI:** 10.1186/s12870-024-04790-6

**Published:** 2024-02-14

**Authors:** Hengzhao Liu, Huijuan Zhou, Hang Ye, Fangdong Gen, Mengfan Lei, Jinhan Li, Wenjun Wei, Zhanlin Liu, Na Hou, Peng Zhao

**Affiliations:** 1 Key Laboratory of Resource Biology and Biotechnology in Western China, Ministry of Education, College of Life Sciences, Northwest University, No. 229 Tabi Rd., Xi’an, 710069 China; 2 Xi’an Botanical Garden of Shaanxi Province (Institute of Botany of Shaanxi Province), Xi’an, 710061 Shaanxi China; 3 Laboratory of National Forestry and Grassland Administration on Biodiversity Conservation in Karst Mountainous Areas of Southwestern China, Guizhou Academy of Forestry, Guiyang, 55005 China

**Keywords:** Metabolism, Walnut, Multiomics, Network analysis, Endopleura

## Abstract

**Background:**

The color of endopleura is a vital factor in determining the economic value and aesthetics appeal of nut. Walnuts (*Juglans*) are a key source of edible nuts, high in proteins, amino acids, lipids, carbohydrates. Walnut had a variety endopleura color as yellow, red, and purple. However, the regulation of walnut endopleura color remains little known.

**Results:**

To understand the process of coloration in endopleura, we performed the integrative analysis of transcriptomes and metabolomes at two developmental stages of walnut endopleura. We obtained total of 4,950 differentially expressed genes (DEGs) and 794 metabolites from walnut endopleura, which are involved in flavonoid and phenolic biosynthesis pathways. The enrichment analysis revealed that the cinnamic acid, coniferyl alcohol, naringenin, and naringenin-7-O-glucoside were important metabolites in the development process of walnut endopleura. Transcriptome and metabolome analyses revealed that the DEGs and differentially regulated metabolites (DRMs) were significantly enriched in flavonoid biosynthesis and phenolic metabolic pathways. Through co-expression analysis, *CHS* (chalcone synthase), *CHI* (chalcone isomerase), *CCR* (cinnamoyl CoA reductase), *CAD* (cinnamyl alcohol dehydrogenase), *COMT* (catechol-Omethyl transferase), and *4CL* (4-coumaroyl: CoA-ligase) may be the key genes that potentially regulate walnut endopleura color in flavonoid biosynthesis and phenolic metabolic pathways.

**Conclusions:**

This study illuminates the metabolic pathways and candidate genes that underlie the endopleura coloration in walnuts, lay the foundation for further study and provides insights into controlling nut’s colour.

**Supplementary Information:**

The online version contains supplementary material available at 10.1186/s12870-024-04790-6.

## Introduction

 Color is a significant factor in determining the commodity value of nuts and fruits and crucial benchmark for assessing their quality [1–4]. With the increasing improvements in people’s living standards, not only are nutrient-rich nuts and fruits required, but also there are higher requirements regarding their appearance and quality [5–7]. The colors of fruits’ skin and flesh are critical factors in determining consumer preference and marketability, not just due to visual effects [8, 9], but also because purple or red colors subjectively imply that they contain beneficial health ingredients [10, 11]. Cultivation of new varieties that look and taste good has become an important goal and direction in nut breeding [5, 8, 12]. Hence, it holds immense theoretical importance and practical application value to explore the characteristics of colorful varieties and comprehend the molecular underpinnings behind the development of fruit color traits [13, 14].

Phenol determination in peel (skin) and seeds has become increasingly important, as it is recognized that these parts are often a source of various phenolic compounds in much higher concentrations than the flesh [3]. Seed coat color is an important characteristic in breeding crops, such as the common bean (*Phaseolus vulgaris*) [15], soybean (*Glycine max*) [16], sesame (*Sesamum indicum*) [17], and *Brassica rapa* [18], but few systematic analyses of seed color have been carried out in woody crops. Walnuts (*Juglans*) are a key edible nut that are high in proteins, amino acids, lipids, and carbohydrates, as well as a variety of trace elements and minerals [19, 20]. Walnuts are a typical food that can be used as a medicine as well as food; it is a medicine listed in the Chinese Pharmacopoeia [21–25]. Its medicinal efficacy is mostly related to the rich polyphenol and flavonoid components, which have antioxidant and immunity-enhancing effects, and has been a hotspot for research and development in recent years [26–28]. The walnut kernel consists of the embryo (seed kernel or kernel) and the endopleura (seed coat, kernel membrane), the walnut endopleura is wrapped in a layer of film on the surface of the kernel [29, 30]. Within the walnut fruit shell, the kernel is surrounded by a protective tan-brown skin referred to as the endopleura. Despite only accounting for 5% of the fruit’s weight, this thin layer is naturally abundant in antioxidant phenolic compounds that aid in safeguarding the kernel against oxidation and rancidity [31]. The walnut endopleura develops from the integument and is generally light yellow, there are specific varieties in which it is purple or bright red [20, 32]. Color change of the walnut endopleura are due to polyphenol oxidation and degradation [33].

Many studies have shown that in the walnut kernel, polyphenols and flavonoids are concentrated in the endopleura, and it is noteworthy that walnuts are particularly rich in polyphenols [34], with a higher polyphenol content than other nuts (e.g., almonds, hazelnuts, and peanuts). The polyphenol metabolites in walnut kernels have a significant impact on walnut quality. The slightly bitter flavor of walnut kernels is related to the phenolic compounds they contain, which are more concentrated in the endopleura than in the kernel [35]. In addition, polyphenols have good antioxidant, anti-inflammatory, anti-mutagenic, and anti-free-radical properties, which can strengthen the immune system [36, 37]. Recently, the composition of the yellow walnut endopleura and the molecular mechanism of its development in the walnut cultivar (‘LinZaoxiang’) have been reported [20]. Interestingly, some walnut cultivars or varieties have red or purple endopleuras [38, 39]; however, very little is known about the gene expression pattern and metabolites in these walnut endopleuras [40, 41]. Significantly high concentrations of anthocyanins were found in red walnut endopleuras compared to light yellow walnut endopleura, in addition to higher contents of calcium, iron, magnesium, and manganese [41]. In addition, significantly higher total phenolic and flavonoid contents were found in yellow endopleuras compared to red endopleura [42]. Thus, a better understanding of both the metabolomic and transcriptomic dynamic profiles of endopleura coloration during fruit maturation will be provide a useful resource for the metabolites related to the endopleura in walnuts.

Research has shown that plant cytochromes are produced by plant phenylpropane metabolites and coumaroyl CoA, through the flavonoid metabolic pathway to produce anthocyanins, flavanols and flavonoids [43]. It has been found that overexpression of *CHS*, *F3H*, *CHI*, *F3’H* and *FLS* genes facilitates polyphenol accumulation in many plants [9, 44–46]. Flavonoids, as secondary metabolites in plants, belong to a class of polyphenolic compounds [47–50]. Flavonoids can be detected in nearly every variety of fruits, vegetables, and other agricultural produce [26, 51]. In addition, various nutritional products contain flavonoids as essential ingredients. The antioxidant activity of flavonoids is their main biological activity and has been extensively studied [52, 53]. Phenolic compounds primarily originate from the phenylpropanoid metabolic pathway. The phenylpropane metabolism is a pivotal secondary plant metabolic pathway, playing a crucial role in various aspects of plant growth, development, and interactions with the environment [19, 54]. Like other signaling pathways in plants, various components of the phenylpropane pathway are subject to a series of fine and complex regulations, which enables plants to more efficiently complete the whole growth cycle and adapt to the variable surrounding environment. The phenylpropane pathway has biological functions via involvement in cellular lignification, cytochrome formation, and rhizome formation processes, among others [55, 56]. Cytochromes are mainly found in plant tissues and organs such as leaves, flowers, fruits and seeds.

Many beneficial acids, tannins and flavonoids are present in the pericarp or endopleura [14, 33, 39]. There is currently a lack of information on which metabolite changes during the development of purple and red skinned walnuts leads to changes in endopleura color. Although we previously investigated the transcriptome dynamics of the developing walnut endopleura [57]. To better understand the different metabolite components and expression changes during the color changes in walnut endopleuras, an analysis using combined transcriptomic and metabolomic methods is needed in purple and red walnuts. In this research, we conducted comparative analysis of both metabolomics and transcriptomics, and conducted a comparative analysis of their differences considering different metabolites and genes to reveal polyphenols and flavonoid compositions and the differences in the endopleura color, with the aim of providing a reference for the study of the chemical composition of walnut endopleuras.

## Materials and methods

### Plant material

 We collected three different varieties of walnuts ‘hongguowuren’ (HGWR), ‘Sajiwuren’ (SJWR), and ‘Sonhewuren’ (SHWR) in two maturity stages required from Panzhou, Guizhou province. The three varieties of walnut trees are all around 50 years old, and they are excellent varieties selected under natural conditions. The collection site was positioned in the middle canopy of walnut trees. During the fruit enlargement period on July 5, 2021 (120 Days after flowering DAF), we collected the fruits of three walnut varieties: SJWR, HGWR, and SHWR. We also collected the fruits during the fruit ripening period, which was the harvest time on September 21, 2021 (harvest time, 165 DAF). For each development stage (120 DAF and 165DAF), and at least 30 representative fruits were sampled from each walnut tree. We then mixed the endopleura from 10 representative fruits of each walnut variety for later sequencing (Table S1). The samples contained three biological duplicates. We measured the chromaticity value of walnut endopleura with 165DAF (Fig. S1B). The L*(brightness), a* (red-green color), and b* (yellow-blue color) color attributes were assessed through reverse transmission mode using a 3700-desktop spectrophotometer. The formula for color difference value is: △E = [(△L*)^2 + (△a*)^2 + (△b*)^2]^0.5. We used the average value of △E for subsequent analysis (Table S2). Samples were promptly chilled using liquid nitrogen and preserved in a freezer at -80 ℃ for amino acid metabolite analysis and transcriptome sequencing. In this study, the *J. sigillata* individual trees identified by Prof. Peng Zhao following the botanical characters leaves, buds, male flowers, female flowers, stem, and fruits. We have been granted authorization to gather the plant specimens by the Guizhou Institute for Forest Resources and Environment, affiliated with Guizhou University. The voucher specimen of *J. sigillata* (deposition accession numbers: NWU20211106, NWU2021107 and NWU2021108) have securely preserved at the Evolutionary Botany Laboratory, Northwest University (Xi’an, Shaanxi, China).

### Extraction of RNA and preparation of cDNA libraries

Total RNA from around 100 mg of frozen walnut endopleuras was extracted using the RNeasy plant mini kit (Qiagen). NanoDrop (Thermo Scientific NanoDrop2000) was used to measure the RNA concentration, and the sample purity was evaluated on 1% agarose gel to evaluate 28 S and 18 S ribosomal RNA bands (28 S/18S ratio). If the ratio of the sample (28 S/18S) exceeds 1.8 and the OD 260/280 ratio is greater than 1.9, it is employed in the process of sequencing. We have evaluated RNA integrity number (RIN) using the RIN algorithm of Agilent Bioanalyzer 2100 system (Agilent RNA 6000 Nano kit, Agilent, catalog numbers 5067 − 1511). Only RNA samples with RIN greater than 7 were passed quality testing. Then, the qualitied RNAs were used to construct cDNA libraries construction (18 cDNA) using a paired-end approach (read length, 150 bp) based on the Illumina HiSeq 2000 platform.

### RNA sequencing data analysis

The initial dataset underwent the removal of adaptors, poly-N sequences, and low-quality reads. Subsequently, a sequential comparison was conducted between the remaining reads and the walnut (*Juglans regia*, ‘Chandler’ v2.0) reference genomic data (http://plants.ensembl.org/Juglans_regia/Info/Index) and set the minimum length of introns, with a default value of 20 using HISAT2 [58], and the comparison rate was found to be greater than 95% for all samples. we used the software DESeq2 [59] for differential expression analysis between sample groups to determine the differential expressed genes (DEGs) among the three varieties. We used |log2Fold Change| > 1, and FDR < 0.05 filters to detect SHWR, SJWR and HGWR. Our assembled genes were searched for homologs using Blastx (E-value < 0.00001) and provided with notes in databases for proteins, such as NR (non-redundant), KOG (Cluster of Orthologous Group), and SwissProt (Swiss Institute of Bioinformatics and Protein Information Resource) databases. The Blast2GO tool [60] was used to assess the functional annotation of GO (Gene ontology, http://www.geneontology.org). Blastall software was used to annotate the KOG and KEGG (Kyoto Encyclopedia of Genes and Genomes) pathways, which were stored in the respective databases [61].

### Profiling of primary metabolites and metabolome extraction

The vacuum freeze-dryer apparatus (Scientz-100 F) is utilized to undergo freeze-drying of the biological samples. The freeze-dried material was subjected to a 1.5-minute pulverization procedure at a frequency of 30 Hz using a mixer mill (MM 400, Retsch) containing zirconia beads. The freeze-dried powder, weighing 100 mg, was combined with a volume of 1.2 mL of 70% methanol solution, stirred for 30 s every 30 min, 6 times, and stored in a refrigerator at 4 °C overnight. The filtered extracts were underwent at 12,000 rpm for 10 min prior to being analyzed using UPLC-MS/MS (www.shimadzu.com.cn). Then we analyzed the sample extract using the UPLC-ESI-MS/MS system (www.appliedbiosystems.com.cn/). The analysis parameters were set as follows: the UPLC analysis was performed using a 1.8 μm Agilent SB-C18 column with dimensions of 2.1 mm*100 mm. The experiment was carried out by implementing a gradient approach, commencing with a mixture consisting of 95% A and only 5% B. In a time frame of 9 min, the procedure achieved a linear gradient of 5% A and 95% B, maintaining the configuration for an additional minute. Following that, the composition was adjusted to 95% content of A and 5.0% content of B for a duration of 1.10 min before being held steady for another 2.9 min. The rate of liquid flow is adjusted to 0.35 ml per minute. Adjust the column oven temperature to 40 °C. The injection volume of injection employed was 4 µL. The ESI-triple quadrupole-linear ion trap (QTRAP)-MS was used for the alternate connection of the effluent. The AB Sciex developed Analyst 1.6.3 software is utilized to manage the instrument. The operating characteristics of the ESI source with the following: the source temperature of the ion source and turbo spray is set at 550 °C; in positive ion mode, the ion injection voltage (IS) is 5500 V, while in negative ion mode it is -4500 V; for gas settings, Ion Source Gas I (GSI), Gas II (GSII), and Curtain Gas (CUR) are adjusted to 50, 60, and 25.0 psi respectively. Collision-activated Dissociation (CAD) is set relatively elevated level. The instrument was tuned and calibrated using a polypropylene glycol solution with concentrations of 100 µmol/L and 10 µmol/L in LIT and QQQ modes respectively. The QQQ scan was performed as a MRM (multiple reaction monitoring) experiment with nitrogen used as the collision gas at medium pressure. By optimizing for decluttering potential (DP) and adjusting collision energy (CE), the DP and CE of a single MRM transition were achieved. The selection of MRM transformations to be monitored in each period is determined by the metabolites that are eluted within that specific time-frame.

### Metabolome data analysis

The prcomp function in R (www.r-project.org) was utilized to conduct unsupervised PCA. The results of HCA (hierarchical cluster analysis) for both samples and metabolites were visualized using heatmaps accompanied by dendrograms, while PCC (pearson correlation coefficients) among eighteen samples were computed using R’s core function and displayed solely as heatmaps [1, 13]. As part of the HCA analysis, metabolite chromatograms were generated to visualize normalized signal intensities. The identification of metabolites that exhibited significant regulation between the groups was conducted by VIP > = 1 and absolute Log2FC (multiple fold change) > 1. The VIP values of OPLS-DA results, including rating charts and permutations, were generated utilizing the R package MetaboAnalystR. Then we used log2Fold Change > = 2, log2Fold Change < = 0.5, and VIP > = 1 to selected differentially accumulated metabolites (DAMs). Approximately 40% of metabolites show significant differences during walnut endopleura development, with flavonoids and phenolic acids serve as the primary discriminative metabolites.

The metabolites that were identified underwent annotation using the KEGG database (http://www.kegg.jp/30kegg/pathway.html). We then analyzed and subjected to enrichment analysis of metabolite sets to determine their relevance using the *p*-value hypergeometric test.

### Correlation analysis of transcriptomic and metabolomic data

We correlated metabolomics with transcriptomic data. We calculated the Pearson correlation coefficients between the DEGs and differentially regulated metabolites (DRMs) using by utilizing the cor function available in R program. Subsequently, both DEGs and metabolites were mapped to the KEGG database for identify of common pathways. In order to identify genes associated with flavonoid synthesis and Phenolic acids, DEGs and DAFs detected at two stages of fruit developmental were selected for comprehensive analysis. We also performed the correlation coefficients and *p*-values between DEGs and DRMs. To further investigate the process of accumulating flavonoids in walnut endopleura, we conducted network interaction analysis on the genes and metabolites associated with the flavonoid pathway. The coefficient method was employed to construct a correlation network diagram linking genes and metabolites involved in common pathways. Utilize Cytoscape version 3.9.1 [62] to visually represent the pertinent network diagram.

### qRT‑PCR verification

The discovered DEG genes’ expression patterns were then investigated using qRT-PCR. The qRT-PCR verification was conducted on endopleura samples obtained from the SJWR during two different developmental periods. Each species/tissue organ had a representation of 3 biological replicates. RNA extraction kits (plant RNA Kit (50) OMEGA, USA) were utilized to isolate total RNA from each sample. The generation of complementary DNA (cDNA) was achieved using the 5× PrimeScript RT Master Mix (Takara) reverse transcriptase. The cDNA mentioned above underwent a 5-fold dilution for use as the template in qRT-PCR. The Bio-Rad CFX96 fluorescent quantitative PCR instrument was utilized for conducting the qRT-PCR experiments., with the fluorescent dye being 2× Plus SYBR real-time PCR mixture (Biotec). The primer design sequences can be found in Table S10. The qRT-PCR findings were assessed using the 2^−ΔΔCT^ approach [63].

## Results

### Quality control of RNA-Seq and transcriptome analysis

**Fig. 1:**
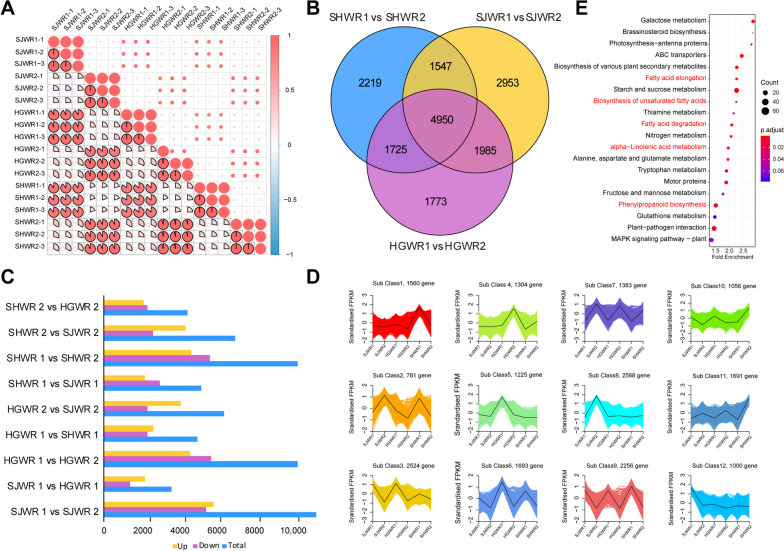
Transcriptome quality and differential transcriptome expression analysis. **A** Correlation analysis of the transcriptome expression profile. **B** Number of differential genes in each group Blue represents the number of total DEGS, yellow represents up-regulation, and purple represents down-regulation. **C** Venn diagram showing the shared and unique DEGs between the three compared groups of peel samples. **D** KEGG analysis of differential genes common to all three groups. **E** K-means cluster analysis of DEGs

We obtained 129.84 Gb clean data, with an average of 48,090,265 clean reads of each accession and 98.16% Q20 ratio and 94.27% Q30per sample (Table S1). Firstly, all the samples from 18 mRNAs inter-sample correlation analysis and heat map analysis were performed on the entire transcriptome data (Fig. 1A, Fig. S2). The results of the heat map indicate that the periods can be distinctly distinguished. The transcriptome sequencing data were evaluated for intra-group reproducibility and inter-group differences among the three walnut varieties. The result indicates that the high dependability of RNA-seq data provides a dependable guarantee for the further data analysis (Fig. 1A, Table S1). These results indicate that changes in gene expression levels at two developmental stages in three different groups of walnut varieties (Fig. 1A). In addition, the correlation heat map also shows a high correlation between three replicates. Also, we found that the gene expression level variations at different developmental stages was greater than the differences between three walnut varieties (Fig. 1A). Total of 4,950 DEGs were found to be common to all three groups (Fig. 1B and C). We performed KEGG analysis of DEGs in three walnut varieties separately. Interestingly, all three sets of DEGs enriched in metabolic pathway, biosynthesis of secondary metabolites pathways, and plant-pathogen interaction. The KEGG analysis of the overlapped 4950 DEGs of three groups at two developmental stages showed that the annotation significant enriched in phenylpropanoid biosynthesis, fatty acid degradation, biosynthesis of unsaturated fatty acids, and alpha-Linolenic acid metabolism pathways (Fig. 1D).

To investigate the gene expression patterns between two developmental stages of July (120DAF, days after flowering) and September (165DAF), we did the K-means clustering analysis of three walnut verities (Fig. 1E). According to the gene expression patterns in endopleuras, the 19,061 genes could be divided into twelve subclasses. Subclass 7 and subclass 9 showed the same gene expression pattern among three walnut varieties, which containing 1,383 genes and 2,256 genes, respectively. Interestingly, all 1,383 genes were upregulated in the second developmental stage (September) compared to first developmental stage (July) of three walnut varieties, while all 2256 genes were downregulated in September compared to July (Fig. 1E). The KEGG analysis showed that subclass 7 genes were enriched in phenylalanine metabolism and starch and sucrose metabolism, and subclass 9 genes were enriched in fatty acid metabolism and fatty acid biosynthesis (Fig. S3).

### Metabolite accumulation of different walnut endopleura

**Fig. 2:**
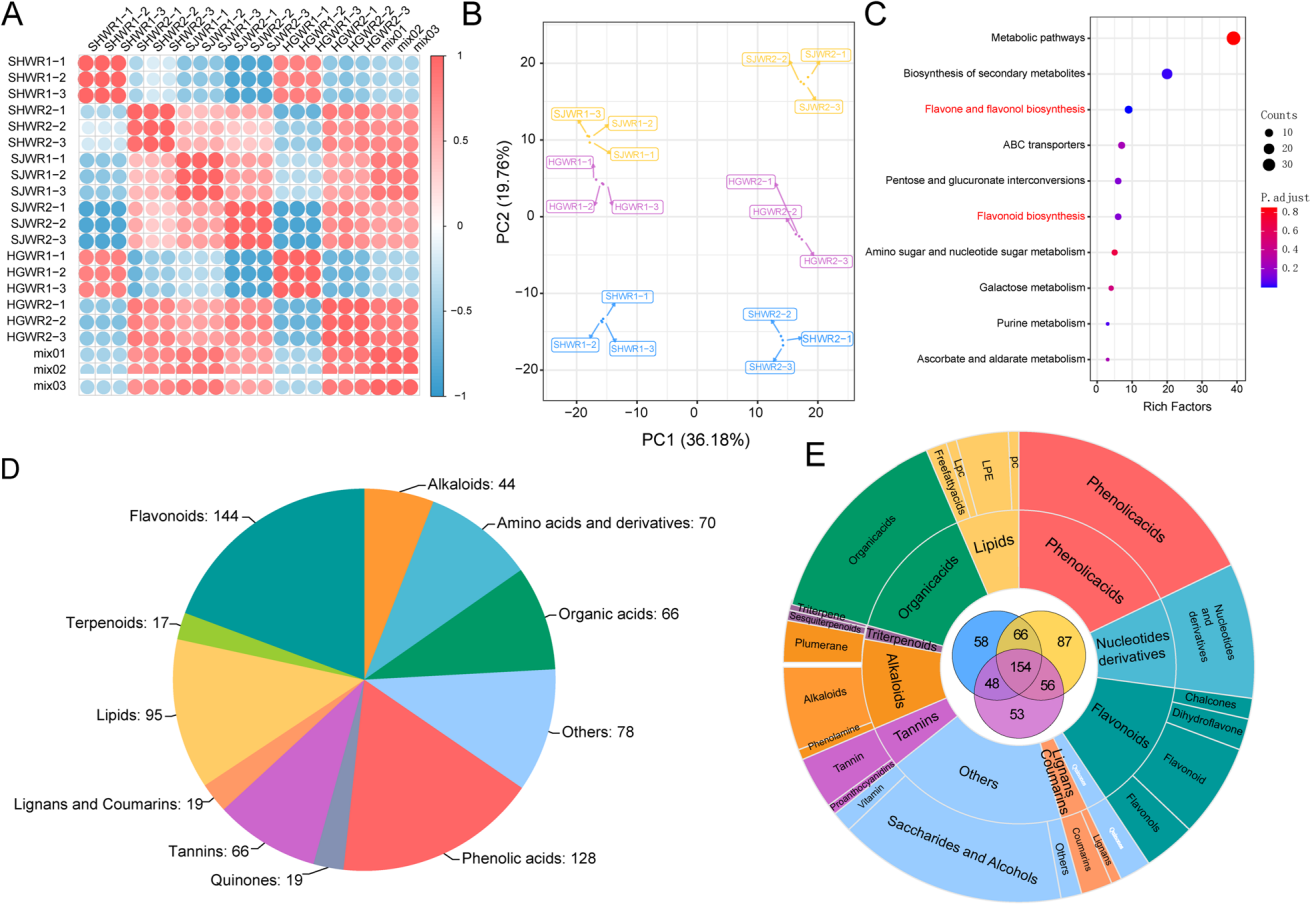
Metabolomics profiles of walnut endopleura. **A** Correlation analysis of HGWR, SJWR, SHWR and mix samples. The color indicates the level of correlation of each sample, from low (blue) to high (red). **B** PCA analysis of different samples. **C** Component analysis of the identified metabolites from walnut endopleura. **D** Classification of differential metabolites common among the three groups of samples. The middle of the circle is a Venn diagram of the differential metabolites of the three groups of samples. **E** Pathway enrichment analysis of common DAMS for three samples

To better explore the color variation of walnut endopleura, we performed metabolomic analysis by LC-MS on three sets of samples. Correlation between samples was analyzed using metabolite concentration data, and the results showed that walnut samples from different periods were clearly distinguishable (Fig. 2A). Intra-group correlation analysis of walnut cortices at different developmental stages also showed high correlation between three biological replicate samples in accordance with the data analysis.

Principal component analysis (PCA) of SJWR, HGWR, and SHWR samples revealed effective separation (Fig. 2B). PC1 and PC2 explained 36.18% and19.76% of the total variance respectively. The PCA results indicate that the differences in growth period are significantly greater than the differences between varieties, suggesting that the variation in walnut endopleura during the developmental period were significant. Furthermore, it is noteworthy that the early gene expression levels of SJWR walnut were significantly different from those of other two walnut varieties during the same period.

We profiled the metabolome of the samples using the widely-targeted metabolomics approach. A total of 794 metabolites were identified (Table S3), which can be categorized into a total of twelve distinct groups (Fig. 2C and Fig. S3). Among that metabolomics, the most abundant compounds are flavonoids (144, 18.1%), and phenolic acids was 16.1% (128), lipids were 11.9% (95), amino acids and derivatives was 8.8% (70), organic acids were 8.4% (66), and tannins was 8.3% (60), respectively. Nucleotides and derivatives (5.9%), Quinones (2.3%), Lignans and Coumarins (2.3%).

Among these 794 metabolites, total of 154 belonged to the differential accumulated metabolites category at two growing stages of common DAMs in walnut endopleura, accounting for 19.39% of the total metabolites detected in 12 categories (Fig. 2; Tables S4 and S5). In these three types of samples, there may be potential metabolites related to the color of the walnut peel. Similarly, we classified these 154 differential metabolites. Among those metabolites, the top five DAMS are Others (16.8%), phenolic acids (16.2%), organic acids (12.9%), flavonoids (12.3%) and amino acids and derivatives (12.3%) (Fig. 2D). These flavonoids could be further categorized as 9 flavonoids, 5 flavonols, 3 dihydroflavones and 2 Chalcones.

A significant proportion of phenolic acids and flavonoids exhibited noticeable variations across differences between growth stages, potentially contributing to the intriguing disparity in color observed in walnut endopleura, we performed KEGG enrichment analysis of these differential metabolites and selected the top ten metabolic pathways. Based on the rich factor, the metabolic pathways were found to be the main source of enrichment for DAMs, flavone and flavanol biosynthesis, and flavonoid biosynthesis pathways (Fig. 2E).

### Differential metabolite analysis of walnut endopleura

**Fig. 3:**
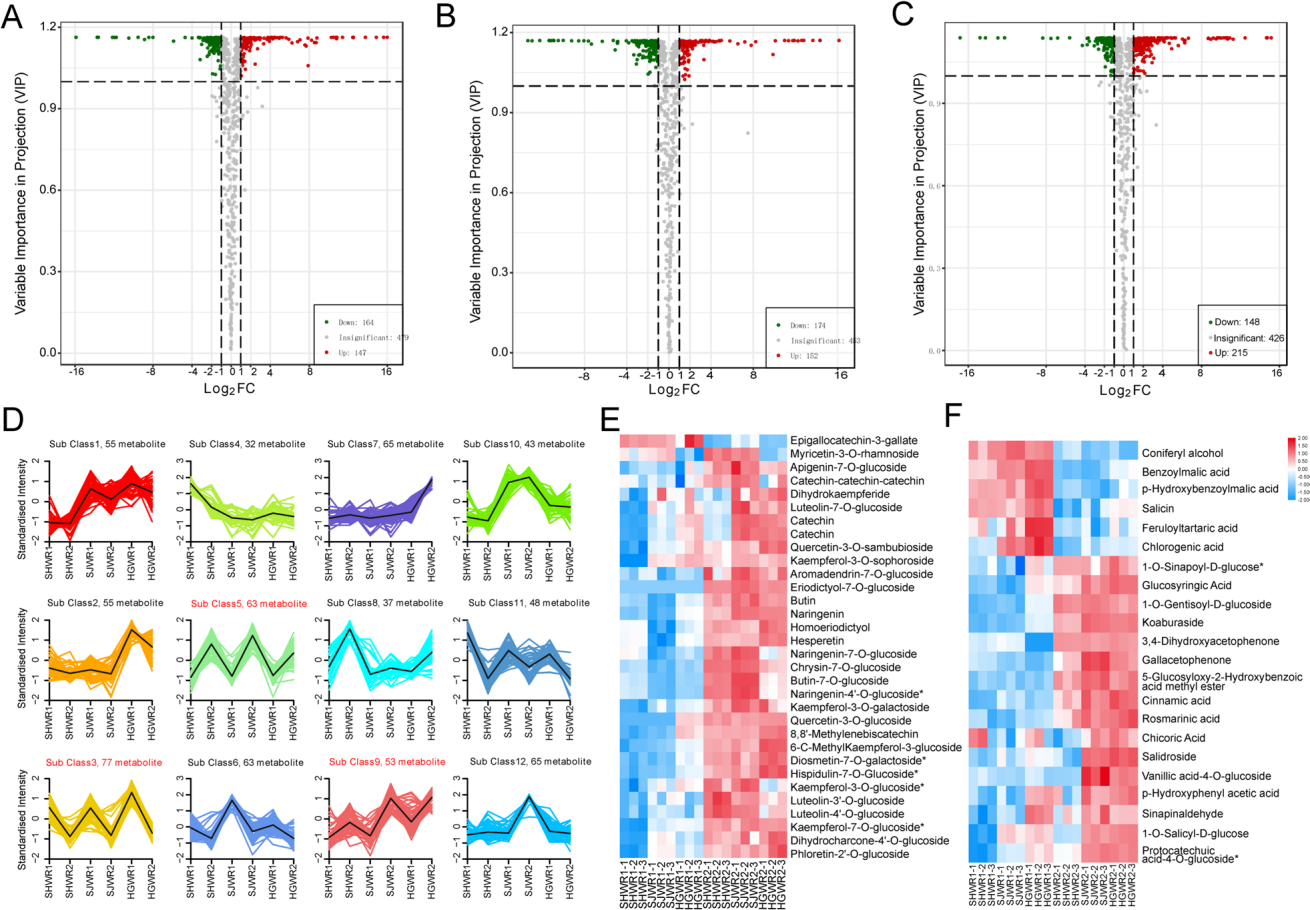
Analysis of the metabolites detected by the metabolome. Volcano plots displaying the up-regulated, down-regulated and no-regulated metabolites between two development stages of walnut endopleuras (**A**, **B**, and **C**). Green dots in the graph represent down-regulated differentially expressed metabolites, red dots represent up-regulated differentially expressed metabolites, and gray represents metabolites that were detected but not significantly different. **D** K-means analysis of differential metabolites. The horizontal coordinate indicates the name of the sample, the vertical coordinate indicates the standardized metabolite relative content, and the sub class represents the metabolite class number with the same change trend. **E** heatmap of differential flavonoid metabolites. **F** heatmap of differential Phenolic acid

As anticipated, a significant quantity of metabolites was differentially accumulated between the comparison samples There were 311 DAMs (147 upregulated, 154 downregulated) in HGWR1 and HGWR2 (Fig. 3A), 326 DAMs (152 upregulated, 174 downregulated) in SHWR1 and SHWR2 (Fig. 3B), and 363 DAMs (215 upregulated, 148 downregulated) in SJWR1 and SJWR2 (Fig. 3C). Notably, flavonoids (12.3%) accounted for the largest proportion of upregulated metabolites, followed by phenolic acids (16.2%).

The key developmental stages of walnut cortex and the dynamic changes of important secondary metabolites accumulation were further discussed. We similarly performed K-means analysis on the differential metabolites and selected the groupings with consistent trends (Fig. 3D). For example, the Sub Class3 (77 metabolites), Sub Class5 (63 metabolites) and Sub Class9 (53 metabolites) (Fig. S4). Among these differentially metabolized species, the number of flavonoids (16.5%) was the largest. In addition, Phenolic acid (11.3%) metabolites also account for a large proportion (Table S6).

Through enrichment analysis of the KEGG pathway, we focused on the main color-related metabolites, including flavonoids and phenolic acids (Fig S5). Finally, a total of 32 flavonoid metabolite species and 22 phenolic acids were identified, all details are provided in Supplementary Table S7. We then performed heat map analysis of flavonoid differential metabolites and phenolic acid differential metabolites. The total content of almost flavonoid and phenolic acid showed an increasing pattern during the two developmental stages of walnut endopleura (Fig. 3E and F). The results of the heat map revealed that flavonoid and phenolic acid DAMS content generally increased in September. Such as, naringenin naringenin-7-O-glucoside, cinnamic acid etc. On the contrary, these differential metabolites (epigallocatechin-3-gallate, coniferyl alcohol etc.) were highly accumulated in July and hardly accumulated in September. This suggests that these metabolites are the critical metabolites responsible for the color change of walnut endopleura.

### Crucial differential metabolites and differential genes in walnut endopleura of different colors in the flavonoid biosynthesis pathway

**Fig. 4:**
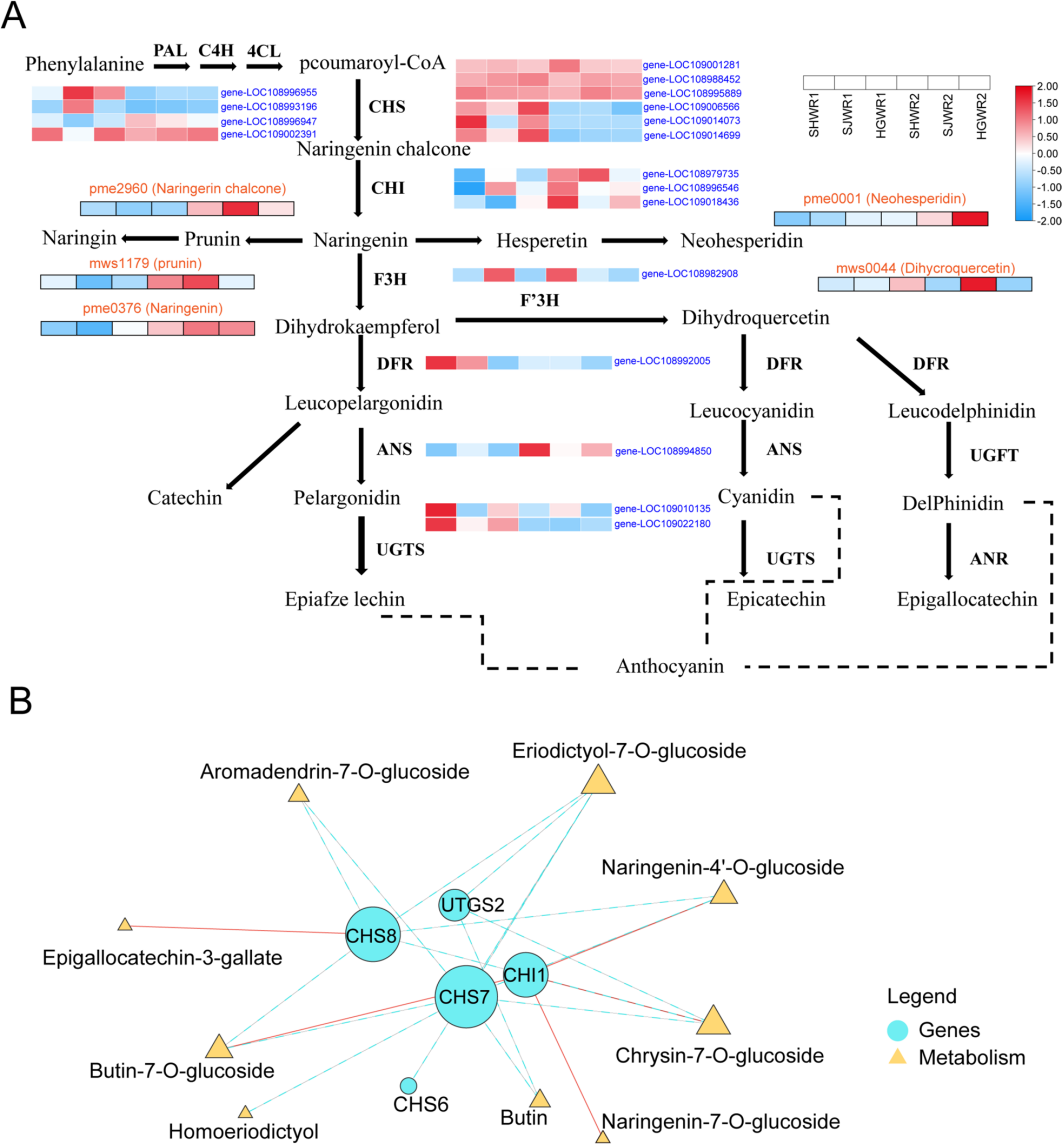
Integrated transcriptomic and metabolomic analysis of the flavonoid biosynthesis pathway. **A** Flavonoid biosynthesis and metabolic pathway of walnut. **B** Interaction Network Analysis of genes and metabolism. Red lines represent positive correlations and blue dashed lines represent negative correlations

Metabolomics analyses showed that flavonoids are the major metabolites in walnut endopleura. Therefore, we focused on the biosynthesis of flavonoids. The emphasis of our research lies in the biosynthesis of flavonoids. According to the known flavonoid biosynthesis pathway, we constructed the flavonoid pathway map with the different gene expression level of enzyme (structural gene) using heat map in walnut endopleura. We identified two classes of genes that showed opposite expression patterns during walnut endopleura coloration. The expression of nine Structural genes (*PAL*, *C4H*, *4CL*, *CHS*, *CHI*, *F3H*, *F3′H*, *DFR*, *ANS*, and *UFGT*) of flavonoid biosynthesis pathway plays a key role in Anthocyanidin biosynthesis. The Structural gene *PAL*, *C4H*, *4CL*, *CHS* and *CHI* are involved in the early enzyme reaction in the process of Flavonoid biosynthesis (Fig. 4A). We found that the expression of early structural genes containing *4CL* genes (gene-LOC108996955, and gene-LOC108993196), *CHS* genes (gene-LOC109006566, gene-LOC109014073, and gene-LOC109014699) were up-regulated in 120 DAF walnut endopleura. The opposite two *4CL* genes (gene-LOC108996947, gene-LOC109002391), three *CHS* genes (gene-LOC109001281, gene-LOC108995889, and gene-LOC108988452), and three *CHI* genes (gene-LOC108979735, gene-LOC108996546, and gene-LOC109018436) were up-regulated in 165 DAF walnut endopleura (Table S8). During naringin catalysis, we found that three important differential metabolites (Naringenin chalcone, Naringenin-7-O-glucoside, and Naringenin) were highly expressed in 165 DAF walnut Endopleura. *F3H* gene can catalyze naringenin into dihydrokaempferol, which is a key precursor and critical branching point of different types of anthocyanins biosynthesis [1]. The expression levels of *F3′H* genes advanced which component of anthocyanins will be synthesized. In addition, the *F3′H* gene catalyzes the conversion of dihydrokaempferol to dihydroquercetin, and we found that the dihydroquercetin was highly expressed in the 165 DAF walnut endopleura stage *DFR* is an important enzyme in the biosynthesis of Anthocyanidin, which has different catalytic capacity for various substrates [1, 64].

To further investigate the process of accumulating flavonoids in walnut endopleura, we performed network interaction analysis of genes and metabolites involved in the flavonoid pathway. Here, we have discovered a *DFR* (gene gene-LOC108992005) that were highly expressed in the 120 DAF walnut endopleura stage and one *ANS* gene (gene-LOC108994850) that were highly expressed in 165 DAF walnut endopleura. *UFGT* and *ANR* are key enzymes in the late stage of Anthocyanidin biosynthesis, and they can catalyze unstable anthocyanidin to anthocyanin. In this process we found two *UGTS* genes (gene-LOC109010135, and gene-LOC109022180) highly expressed in the 120 DAF walnut endopleura stage. We also plotted the correlation network of the DEGs and DAMs (Fig. 4B). Through relevant network regulation analysis, it was found that Naringenin-7-O-glucoside, Naringenin-4’-O-glucoside, Butin-7-O-glucoside, Chrysin-7-O-glucoside and Epigallocatechin-3-gallate showed a positive correlation with *CHI1* (gene-LOC108981066) and *CHS8* (gene-LOC109014699) (Fig S6). In contrast, the Metabolisms (Aromadendrin-7-O-glucoside, Eriodictyol-7-O-glucoside, Naringenin-4’-O-glucoside, Butin-7-O-glucoside, Homoeriodictyol, Butin, Chrysin-7-O-glucoside) exhibit a negative correlation in *CHS8(gene-LOC109014699)*, *CHS7* (gene-LOC109014073) *CHS6* (gene-LOC109006566) *CHI1*(gene-LOC108981066) and *UTGTS2*. These results suggested that these five genes may play key roles in the synthesis of Anthocyanidin in walnut endopleura.

### Transcriptome and metabolome of the acid pathway with different endopleura colors

**Fig. 5:**
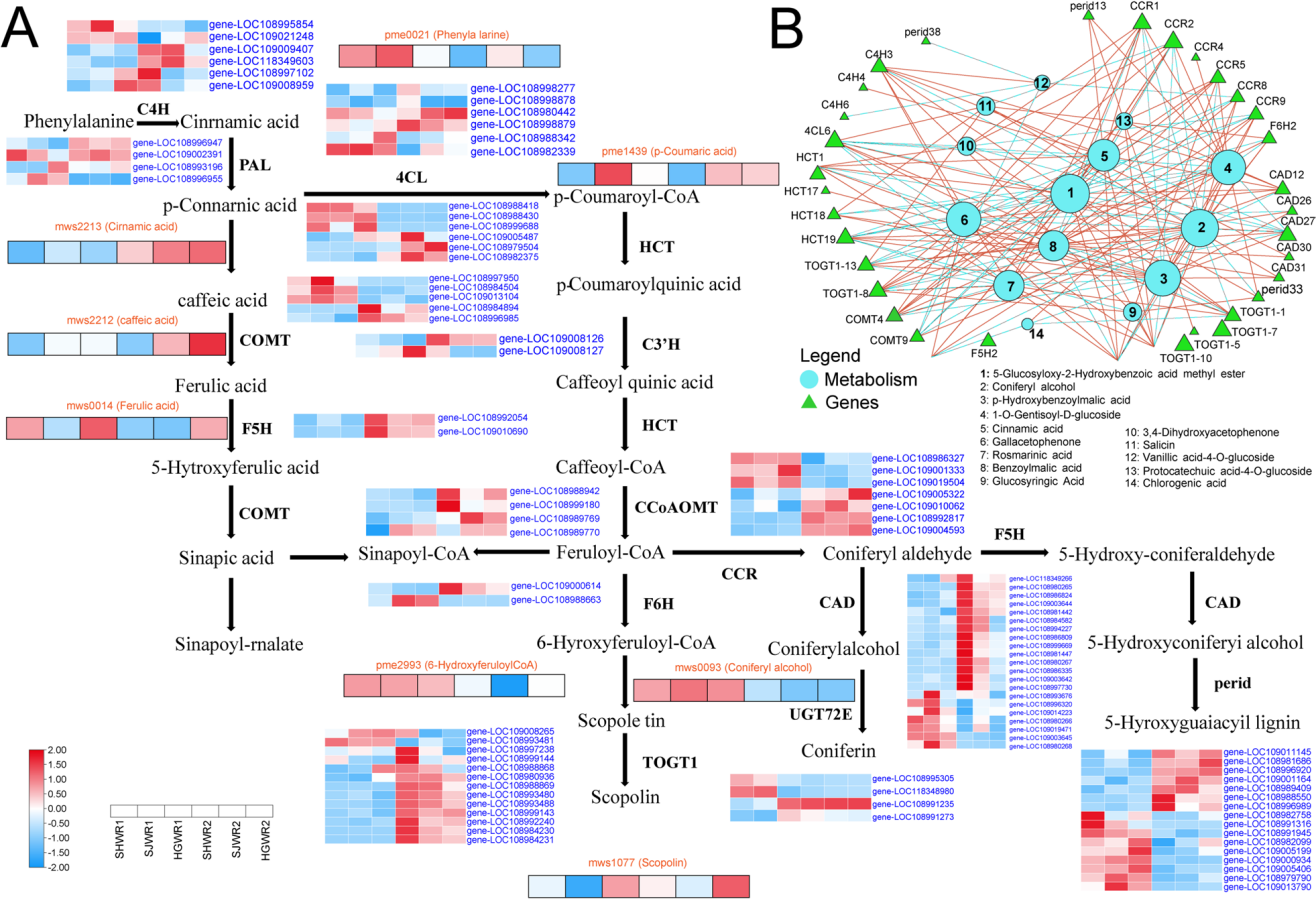
Phenolic metabolic pathways and associated network diagrams. **A** Modulation of phenolic biosynthesis pathway genes during walnut Endopleura. **B** Co-expression analysis of structural genes and metabolites of the phenolic acid biosynthetic pathway in walnut at different developmental stages. Blue nodes represent metabolites and green nodes represent genes. The solid red line represents a positive correlation and the dashed green line shows a negative correlation

Although phenolic compounds have no known nutritional function, their good antioxidant properties make phenolic compounds potentially responsible for protecting walnut kernels from oxidation of valuable fatty acids, thus protecting the intrinsic quality of walnut kernels. We found phenolic metabolites to be among the most abundant metabolite species among the differential metabolites, suggesting that a multigene synergistic model of phenolic biosynthesis may exist. Based on the DAMs and DEGs identified in this study, we mapped schematically to illustrate the pathways of phenolic compound synthesis in walnut. Combined with previous studies, we identified 11 genes (*C4H*, *PAL*, *4CL*, *HCT*, *C3’H*, *COMT*, *F5H*, *CCoAOMT*, *CCR CAD*, *UGT72E*) expressed in the phenolic metabolic pathway. In the phenolic metabolism pathway (Fig. 5A), we found that two *C4H* genes (gene-LOC108995854, gene-LOC109021248), two *PAL* genes (gene-LOC108993196, gene-LOC109002391), two 4 cl genes (gene-LOC108988342, gene-LOC108982339), three *HCT* genes (gene-LOC108988418, gene-LOC108988430 and gene-LOC108999688), one *C3’H* genes (gene-LOC109008127), three *CCoAOMT* genes (gene-LOC108986327, gene-LOC109001333 and gene-LOC109019504), one *F6H* gene (gene-LOC108988663), two *TOGT1* genes (gene-LOC109008265, gene-LOC108993481), seven *CAD* genes (gene-LOC108993676, gene-LOC108996320, gene-LOC109014223, gene-LOC108980266, gene-LOC109019471, gene-LOC109003645 and gene-LOC108980268), two *UGT72E* genes (gene-LOC108995305, gene-LOC118348980), and nine *perid* genes (gene-LOC108982758, gene-LOC108991316, gene-LOC108991945, gene-LOC108982099, gene-LOC109005199, gene-LOC109000934, gene-LOC109005406, gene-LOC108979790 and gene-LOC109013790) were highly expressed in the 120 DAF walnut endopleura stage. In contrast to these genes, which are highly expressed in the 165 DAF walnut endopleura stage, two *C4H* genes (gene-LOC109009407, gene-LOC118349603), two *PAL* genes (gene-LOC108996947, gene-LOC109002391), two *4CL* genes (gene-LOC108980442, gene-LOC108998879), three *HCT* genes (gene-LOC109005487, gene-LOC108979504 and gene-LOC108982375), two *COMT* genes (gene-LOC108984894, gene-LOC108996985), one *C3’H* gene (gene-LOC109008126), two *F5H* genes (gene-LOC108992054, gene-LOC109010690), four CCoAOMT genes (gene-LOC109005322, gene-LOC109010062, gene-LOC108992817 and gene-LOC109004593), one *F6H* gene (gene-LOC109000614), eight *TOGT1* genes (gene-LOC108980936, gene-LOC108988869, gene-LOC108993480, gene-LOC108993488, gene-LOC108999143, gene-LOC108992240, gene-LOC108984230 and gene-LOC108984231), one *UGT72E* genes (gene-LOC108991235), five *CAD* genes (gene-LOC118349266, gene-LOC108980265, gene-LOC108986824, gene-LOC109003644 and gene-LOC108981442) and seven *perid* genes (gene-LOC109011145, gene-LOC108981686, gene-LOC108996920, gene-LOC109001164, gene-LOC108989409, gene-LOC108988550 and gene-LOC108996989) were highly expressed in 165 DAF walnut endopleura (Table S9). Additionally, most of the genes for *CAD* and perid expressed were found at higher levels in 165 DAF (Fig. 5). Moreover, Cinnamic acid and caffeic acid were highly expressed at 165 DAF, while Coniferyl alcohol and Scopoletin were highly expressed at 120 DAF. Furthermore, we mapped the network of differential genes associated with differential metabolites in the phenolic metabolic pathway (Fig. 5B).

During the development of walnut endopleura, the metabolites that differ significantly interacted with the results of the network of differential genes. Interestingly, the results showed that mainly 14 metabolites (5-Glucosyloxy-2-Hydroxybenzoic acid methyl ester, Coniferyl alcohol, p-Hydroxybenzoylmalic acid, 1-O-Gentisoyl-D-glucoside, Cinnamic acid, Gallacetophenone, Rosmarinic acid and Benzoylmalic acid etc.) The results showed that the accumulation of metabolites regulated by the expression of structural genes involved in the synthesis of 10 enzymes (*CCR*, *F6H*, *CAD*, *perid*, *TOGT*, *F5H*, *COMT*, *HCT*, *4CL*, and *C4H*) was involved in the pathways related to phenol metabolism.

### qRT-PCR validation of RNA-seq data

**Fig. 6:**
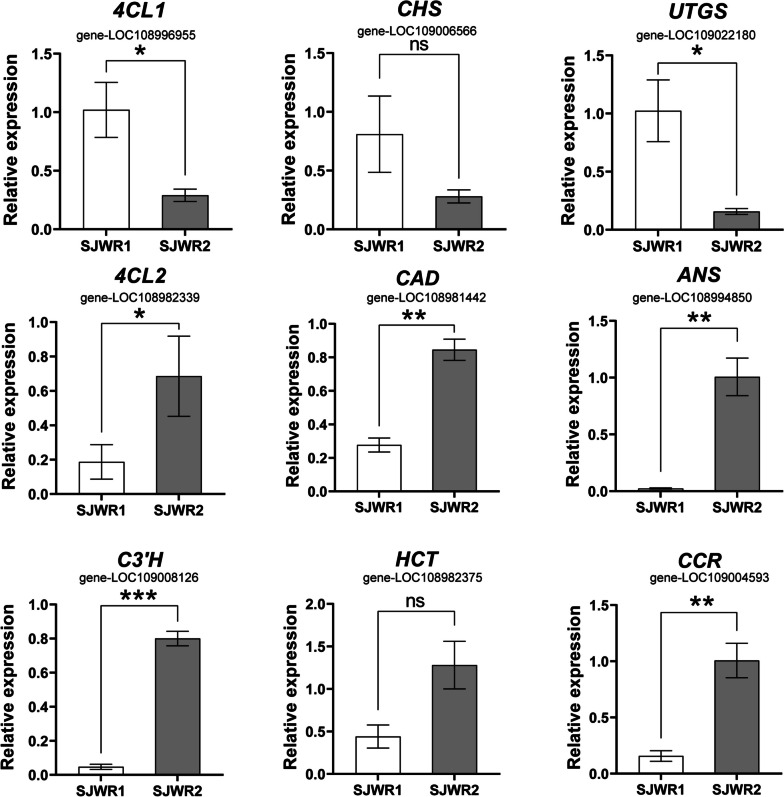
qRT-PCR validation of differential expression. ns = no significant different, *=*p* < 0.05, **=*p* < 0.01, ***=*p* < 0.001

To ensure the precision and reproducibility of the transcriptome analysis findings, we selected and confirmed 9 structural genes from the flavonoid and acid pathways using qRT-PCR. The qRT-PCR analysis showed the same expression trends as the RNA-Seq data for all 9 genes (Table S10). The correlation analysis of the metabolome and transcriptome profiles indicates that all the gathered data is highly reliable (Fig. 6).

According to the qRT-PCR result, three genes (gene-LOC108996955, gene-LOC109006566, gene-LOC109022180) were highly expressed in the 120 DAF walnut endopleura stage. Seven genes (gene-LOC108994850, gene-LOC108996947, gene-LOC109008126, gene-LOC108982375, gene-LOC109004593, gene-LOC108982339, gene-LOC108981442) were highly expressed in n the 165 DAF walnut endopleura stage.

## Discussion

Endopleura color is a vital factor for determining the economic value and aesthetic appeal of nuts and in crop breeding. Very little systematic analyses of endopleura color have been carried out in woody crops [19, 26]. Walnuts (*Juglans*) are a key ebible nut that are high in proteins, amino acids, lipids, and carbohydrates, as well as a variety of trace elements and minerals [20, 25]. In this study, we investigated transcriptomes and metabolomes profiles during two endopleura developmental stages of walnuts. The results illuminate the metabolic pathways and candidate genes that underlie the endopleura coloration in walnuts.

### Gene expression profiles related to flavonoids and anthocyanins during two endopleura developmental stages in walnuts

Flavonoids/anthocyanin metabolite biosynthesis is governed by structural genes, which are subject to regulation by transcription factors [65]. Many studies have been focused on the gene expression levels in flavonoids/anthocyanin biosynthesis pathway, it was found that high expressions of *PAL*, *C4H* and *4CH* ensured the production of p-coumaroyl-CoA in pepper fruits [7]. *PAL*, *C4H*, and *4CH* structure genes are expressed highly in *Cinnamomum camphora*, pineapple peel, and snow chrysanthemum [1, 66, 67]. In this study, we observed that the majority of structural genes in the initial stages of the flavonoid biosynthesis pathway were highly active at 120 DAF, but were gradually suppressed by 165 DAF. We further identified that Naringenin chalcone, prunin, and Naringenin were highly expressed at 165DAF. Based on a network interaction analysis, we found that the differently expressed genes *CHS*, *CHI* and *UTGS* were strongly associated with differential metabolites (Butin-7-O-glucoside, Naringenin chalcone and Eriodictyol-7-O-glucoside). These results were also found in applse and grapes [43, 68]. The expression levels of *FLS* (MD08G1121600) and *CHI* (MD01G1167300) were significantly reduced in the overexpressed apple skin. In the grape berry pericarp, UGT88A1 is a key structural gene that is negatively correlated with some flavonoids. The *PAL*, *C4H*, *4CL*, *CHS*, *CHI*, *F3H*, *LDOX*, and *ANR* were highly expressed in the maturity development stage in yellow walnut endopleura [33]. Most of the *CHS* (Jr01G10656, Jr02G10304), *F3’H* (Jr07G12902) and *C4H* (Jr13G11700) genes involved in polyphenol synthesis are highly expressed during the walnut ripening stage(p5).

### Metabolite differences during two endopleura developmental stages in walnuts

The composition of plant metabolites is intricate and typically composed of primary and secondary metabolites [7, 13]. Genetic variations result in different metabolic compositions between species, particularly in respect to secondary metabolites [48, 69, 70]. The key to researching and utilizing a species is to comprehend its metabolite status. Several studies have revealed that walnuts contain a high concentration of secondary metabolites, particularly polyphenols and flavonoids [19, 71, 72]. The metabolic profiles of purple-skinned walnut cultivars have not been extensively studied. Although the weight of the walnut endocarp is relatively light, it serves as the primary source of flavonoids in walnut embryos and features exceedingly high contents of several key components. The walnut endocarp is significantly rich in flavonoid substances, especially within the membrane, which gradually increase with the development of the endocarp (Fig. 2). An analysis of differentially accumulated metabolites in the two periods indicated that flavonoids and phenols regulate the transition of the walnut endopleura to a purple color. Out of 70 upregulated metabolites, 2 unique anthocyanins (3-O-glucoside anthocyanidin and O-syringic acid) were found only in RP longan.

Plant pigmentation is mainly regulated by flavonoids [34, 45, 57]. Although the walnut endopleura has a weaker physical protective barrier function compared to the outer shell, it undeniably has a strong chemical defensive function that protects the walnut kernel. This protective function mainly arises from a high concentration of diverse phytochemicals [20, 33]. Walnuts contain a significant amount of lipoprotein-bound antioxidants. In addition, the walnut endopleura is the main source of phenolic compounds [72, 73].

Due to the regulation of gene expression by multiple factors, it is challenging to determine metabolite accumulation solely by analyzing transcriptome expression levels. Therefore, an integrated analysis of metabolomics and transcriptomics is an effective method for revealing the interactions between gene expression and metabolite differences and is a very popular method in fruit and seed development studies [1, 4, 64, 68]. Transcription factors (*R2R3-MYB*, *bHLH*) and a repeat protein (WD40) are the main flavonoid regulatory factors in the jujube leaves. In pineapple peel, positive regulators of anthocyanin accumulation are AcMYB12 and AcHOX21. We investigated the differential metabolites and genes that showed significant enrichment in phenylpropane biosynthesis, flavonoid biosynthesis, and two metabolic pathways during the development of purple-skinned walnut fruits via a transcriptome metabolome combination analysis.

### Differential gene expression and metabolite accumulation of the acid pathway

Phenylpropane biosynthesis forms the foundation of the secondary metabolism of polyphenols and flavonoids [33, 63, 74]. Its phenolic intermediates and key enzymes serve to determine the direction of the branching metabolism [75]. Numerous studies have demonstrated that the plant phenolic metabolism is a multifaceted regulatory network that encompasses a plethora of genes [19, 58, 68]. The phenolic metabolism is also intricately associated with gene expressions, including *C4H*, *PAL*, *CHS*, *4CL*, *CAD*, and *F5H* expression [44, 74]. In the present study, the gene expression analysis indicated the involvement of a total of 164 genes in the phenolic metabolism during walnut fruit development. Through the phenolic acid metabolic pathway, it was observed that the *PAL*, *HCT*, *CCoAOMT*, *HCT*, *CCR*, *CAD*, and *perid* genes involved in the phenolic metabolism exhibit varying expression levels at different stages of walnut fruit development (Fig. 5), which was also found in phenolic metabolism. Anthocyanins (3-O-6″-malonylglucoside, cyanidin 3-O-glucoside, and cyanidin O-syringic acid) were uniquely contained in the longan (*Dimocarpus longan* Lour.) seed coat after coloring the red pericarp (RP) longan. Moreover, the DEGs were significantly enhance in flavonoid and flavone pathways. More importantly, *F3′H* and *F3′5′H* structural genes play a significant role in the synthesis of anthocyanin components [44]. Furthermore, *COMT* in grape berry skin was significantly correlated with seven phenolic acid compounds. The flavonoid and phenolic acid contents in V-shaped grape skins are significantly higher than those in vertical shoot-positioned and T-shaped crown grape skins. Additionally, *CHI*, *UGT*, and *CCOMT* are significantly associated with 15 flavonoids (e.g., neochlorogenic acid and gentisic acid) [57]. Plant phenolic substances, particularly flavonoids, are primarily derived from the metabolism of the phenylpropanoid pathway and are controlled by related enzyme activities [10, 76, 77]. Both *PAL* and *ANS* expression levels increased in August [4]; there is a high rate of phenolic compound synthesis. In this study, it was observed that the expression level of *ANS* was higher at 165DAF than120DAF. The red walnut endopleura color might be due to the presence of four derivatives of cyanidin and delphinidin hexosides [4]. P-Coumaroyl-CoA, an essential intermediate, also contributes to the production of flavonoids, phenolic acids, and lignin and is highly expressed at 165DAF. We found that two *PAL* genes (gene-LOC108996947 and gene-LOC109002391) indirectly affect the accumulation of p-Coumaroyl-CoA, thereby regulating changes in the walnut endopleura color.

Recent studies have suggested that CsCCoAOMT1 prefers flavonoids over caffeoyl CoA and esculetin. This enzyme has a strong preference for quercetin (flavanol) and flavones, and effectively methylated a large number of 6-, 7-8-, and 3’- OH flavonoids with adjacent hydroxyl groups [76]. Additionally, the biological functions of the phenylpropanoid metabolism pathway in plants are linked to processes such as cell lignification, cytochrome formation, and root nodule formation [47]. Lignin serves a vital purpose in plant growth and development. Nonetheless, the study of genetic modification to decrease plant lignin content persists [68]. Nevertheless, insufficient research exists on the regulation of CAD genes, particularly in dicotyledonous plant species. Our study indicates that the lignification process occurs during walnut maturation. Can CAD enzymes also regulate transformations of the walnut endopleura? Interestingly, we discovered that *CAD*, a crucial enzyme in the lignin metabolism pathway, demonstrates high expression levels at 165DAF during the metabolic process. We hypothesize that these genes influence walnut endopleura lignification and lead to alterations in endopleura color. Additionally, walnut ripening impacts certain peroxidase genes and alters the skin color. Research indicates that peroxidase genes play a role in anthocyanin degradation in diverse plant species, including grapes and lychees [43, 78, 79]. Consequently, we postulate that the suppression of peroxidase genes in mature walnut endopleura may prevent the breakdown of anthocyanins, resulting in a purple walnut endopleura.

## Conclusion

In present study, a comparative analysis of metabolites at two different stages was performed using the endopleura of light purple walnuts as material and combining transcriptomics and metabolomics analysis. In the endotesta of walnuts, a total of 4,950 differentially expressed genes (DEGs) and 794 differentially accumulated metabolites (DAMs) were identified at two different developmental stages from three walnut varieties. A comparison of the metabolites in the seed coat of purple walnuts at different stages was then performed, revealing the regulatory effect of enzymes in the phenolic and flavonoid pathways (*CHS*, *CHI*, *UTGS*, *CCR*, *CAD*, *Perid* and *4CL*) on the color of the walnut endopleura. We found that the DEGS *CHI* (gene-LOC108979735, gene-LOC108994850) and *ANS* (gene-LOC108994850) was consistent with the differential expression of flavonoid metabolites (Naringenin chalcone and Naringenin). The differences in the phenolic metabolites (cinnamic acid, Coniferyl alcoho) and the DEGS *4CL* (gene-LOC108982339) and *COMT* (gene-LOC109004593) are consistent. Subsequently, qRT-PCR was used to confirm the expression patterns of these important genes. *CAD*, *CHI* and *CHS* transcription factors may be candidate regulator of the synthesis of phenolic metabolites in walnut seed coat. The results of the study suggest that changes in the expression levels of genes regulating phenolic and flavonoid synthesis have an impact on the formation of walnut quality and the accumulation of metabolites associated with cell membrane color throughout walnut development. Consequently, this study can be used as a reference point for functional research and subsequent development and exploitation of walnut endotesta.

### Supplementary Information

**Additional file 1:  Table S1.** Statistics of RNA-seq data for all samples. **Table S2.** The chromaticity values of three walnut varieties in 165 DAF. **Table S3.** All metabolites of two stages of three walnut cultivars endopleuras. **Table S4.** Differential metabolites in each group. **Table S5.** Three sets of common differential metabolites. **Table S6.** Metabolites from Clusters (3, 5, and 9) in metabolomic k-means analysis. **Table S7.** Differential metabolites and genes in the flavonoid metabolism pathway. **Table S8.** Differential metabolites and genes in the flavonoid metabolism pathway. **Table S9.** Differential metabolites and genes in the Phenolic metabolism pathway. **Table S10.** Primers for the qRT-PCR experiment.

**Additional file 2:** **Fig. S1. **The morphology and color difference of three walnut varieties. **Fig. S2. **Cluster heat map of differentially expressed genes (DEGs) among three varieties of walnuts. **Fig. S3. **Analysis of the KEGG pathway and GO enrichment of differentially expressed genes in each group of walnuts. **Fig. S4. **Enrichment analysis of cluster 7 and cluster 9 in K-means. **Fig. S5.** Enrichment analysis of metabolites and distribution patterns of the top 10 differential metabolites in three walnut cultivars. **Fig. S6. **Differential expression of genes and metabolites at two walnut endopleura stages.

## Data Availability

Metabolomics data will be made available on request. Transcriptomics data of two developmental stages of walnut endopleura of three cultivars in this study have been deposited in the NCBI accession number: PRJNA1019965.

## Abbreviations

DAF: Days after flowering
KEGG: Kyoto Encyclopedia of Genes and Genomes
HCA: Hierarchical cluster analysis
PCC: Pearson correlation coefficients
DAMs: Differentially accumulated metabolites
DEGs: Differentially expressed genes
DRMs: Differentially regulated metabolites
CHS: Chalcone synthase
CHI: Chalcone isomerase
CCR: Cinnamoyl CoA reductase
CAD: Cinnamyl alcohol dehydrogenase
COMT: Caffeic acid 3-O-methyltransferase
4CL: 4-coumaroyl: CoA-ligase
F3'H: Flavanone 3'-hydroxylase
FLS: Flavonol synthase
F3H: Flavanone 3-hydroxylase
PAL: Phenylalanine ammonia-lyase
ANS: Anthocyanidin Synthase
C4H: Cinnamate 4-hydroxylase
UFGT: Flavonoid-3-O-glucosyltransferase
UGTS: UDP-glycosyltransferases
DFR: Dihydro-flavonol 4-reductase
C3’H: p-coumaroyl quinate/shikimate 3'-hydroxylase
COMT: Catechol-Omethyl transferase
F5H: Ferulate 5-hydroxylase
F6H: Flavone 6-hydroxylase
CCoAOMT: Caffeoyl-CoA-O-methyltransferase
