# Supplementary material for: Integrated metabolomic and transcriptomic dynamic profiles of endopleura coloration during fruit maturation in three walnut cultivars
Source: BMC Plant Biol. 2024 Feb 14;24:109. doi: 10.1186/s12870-024-04790-6 (PMC10865529; doi:10.1186/s12870-024-04790-6)
Supplement: Supplementary file 2 — Additional file 2: Fig. S1. The morphology and color difference of three walnut varieties. Fig. S2. Cluster heat map of differentially expressed genes (DEGs) among three varieties of walnuts. Fig. S3. Analysis of the KEGG pathway and GO enrichment of differentially expressed genes in each group of walnuts. Fig. S4. Enrichment analysis of cluster 7 and cluster 9 in K-means. Fig. S5. Enrichment analysis of metabolites and distribution patterns of the top 10 differential metabolites in three walnut cultivars. Fig. S6. Differential expression of genes and metabolites at two walnut endopleura stages. [file 12870_2024_4790_MOESM2_ESM.docx]

**Supplementary information**

**
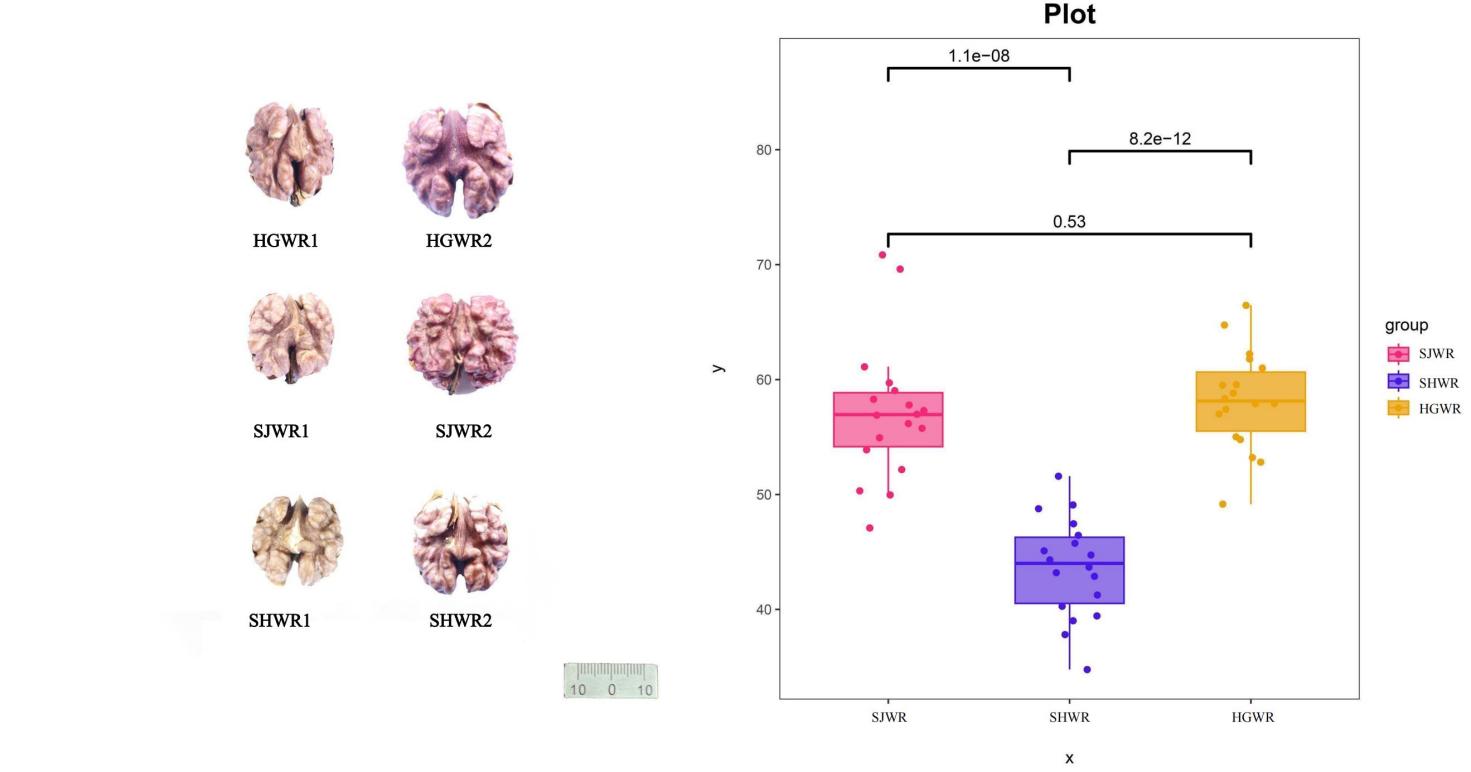
**

**Fig. S1. The morphology and color difference of three walnut varieties.** (A) Morphology of walnut seed coat at different stages. DAF: Days after flowering. (B). The color difference of three walnut endopleura in 165 DAF period. Hongguowenren (HGWR), Sangjiwenren (SJWR), and Sonhewuren (SHWR). DAF = Days after flowering. ns indicate that no significant, * indicate that *P* <0.05, ** indicate that *P* <0.01, *** indicate that *P* <0.001.


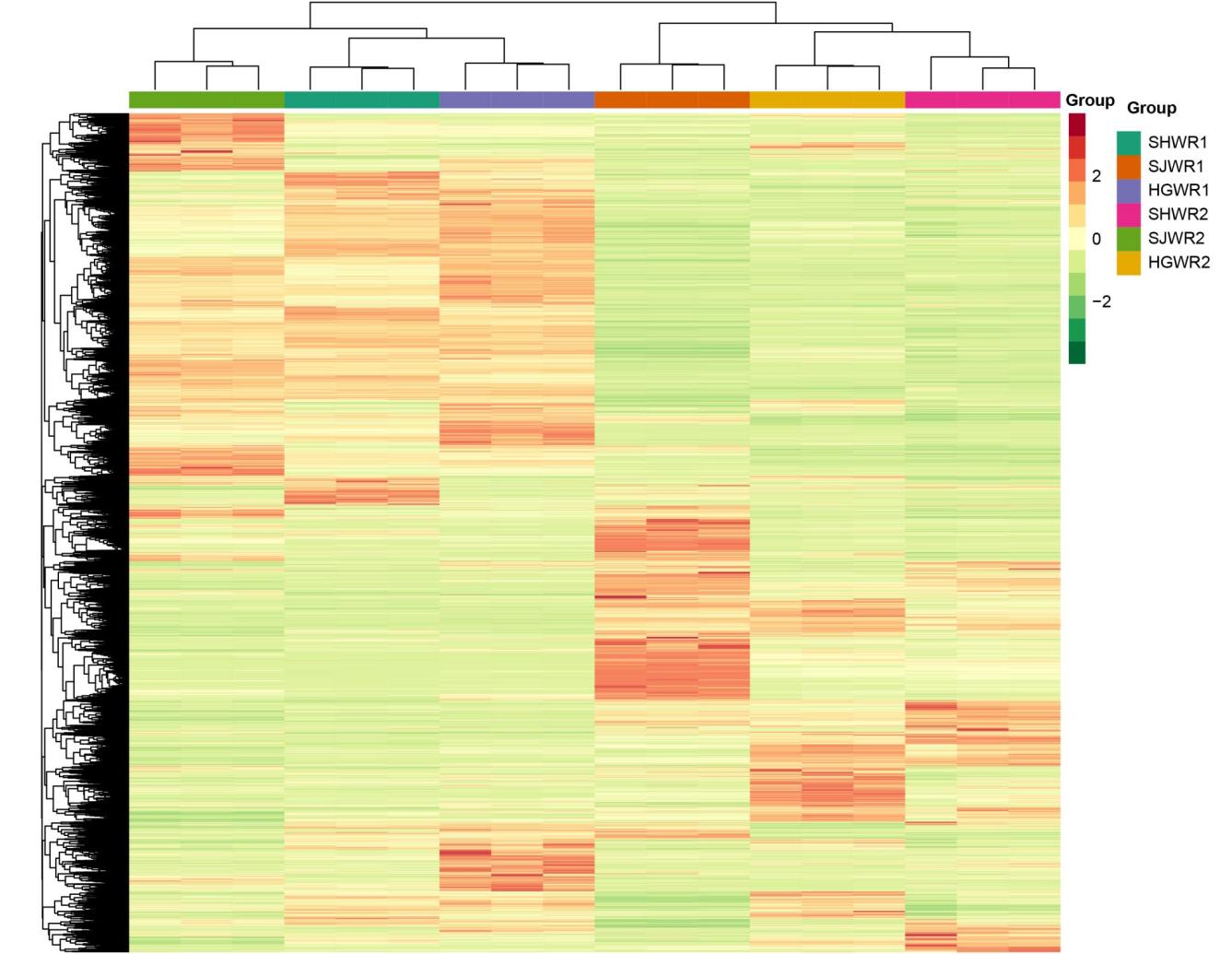


**Fig. S2. Cluster heat map of differentially expressed genes (DEGs) among three varieties of walnuts.**


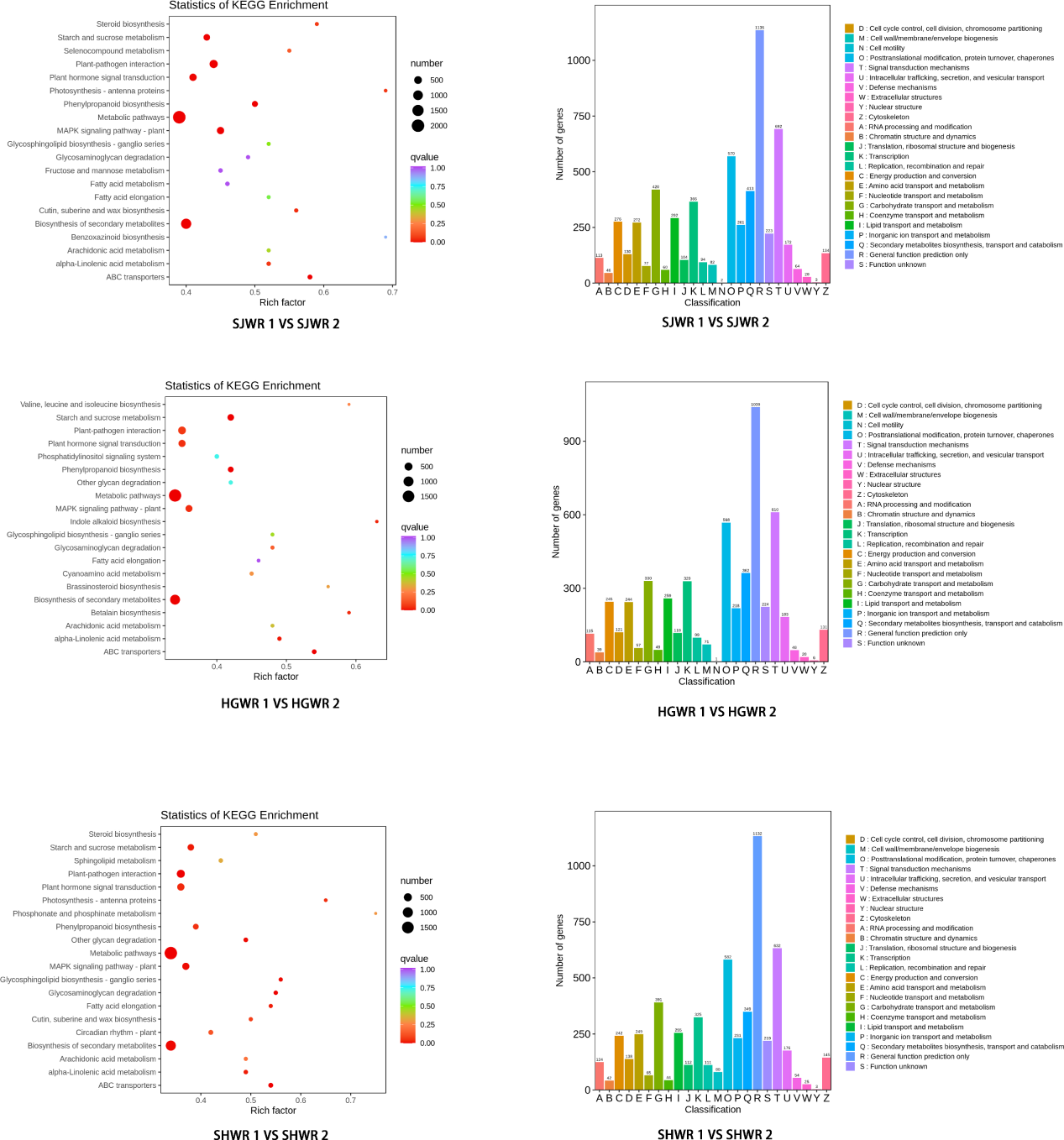


**Fig. S3. Analysis of the KEGG pathway and GO enrichment of differentially expressed genes in each group of walnuts.**


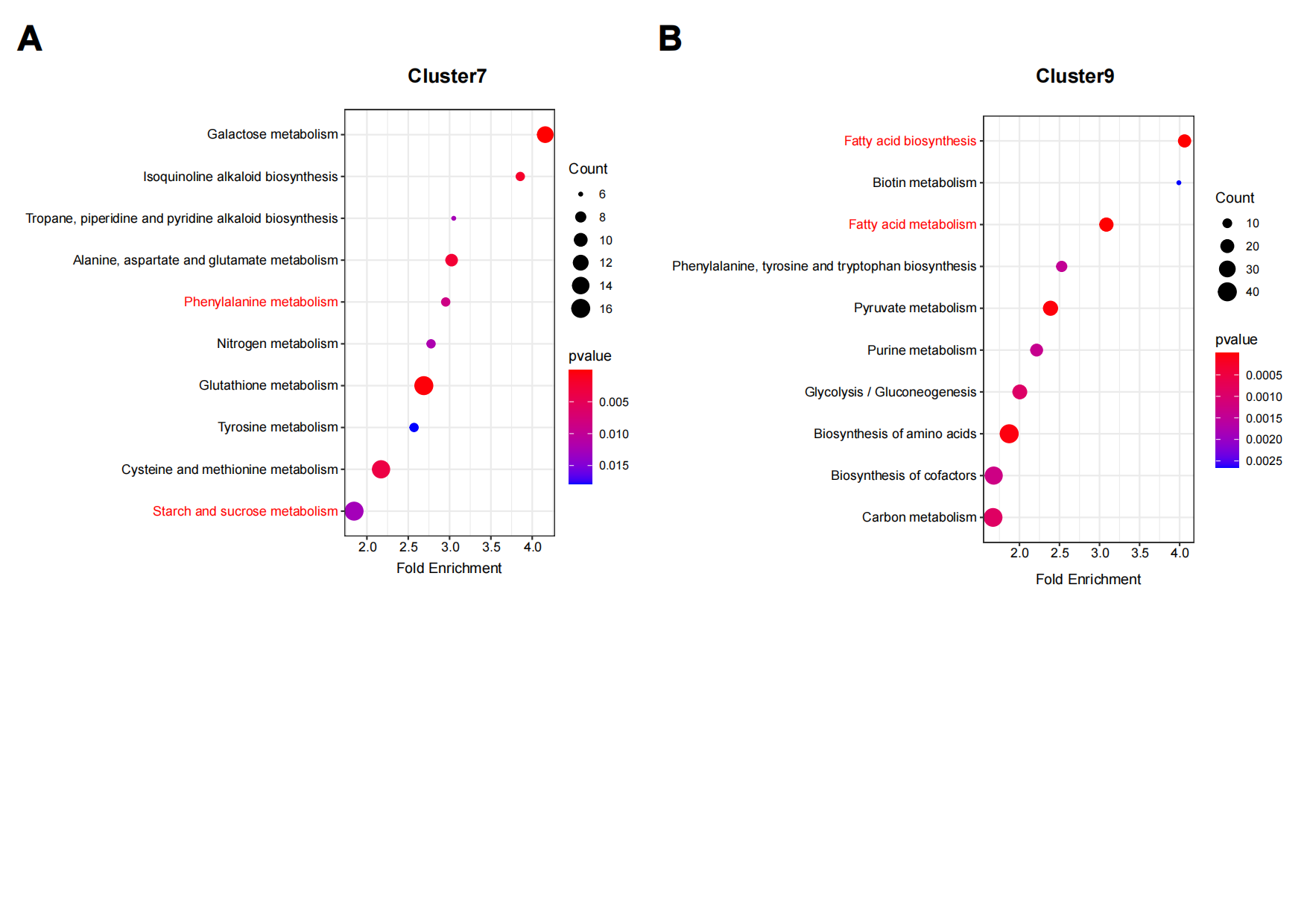


**Fig. S4. Enrichment analysis of cluster 7 and cluster 9 in K-means.** The size is the quantity, the redder the color, the smaller the P-value.


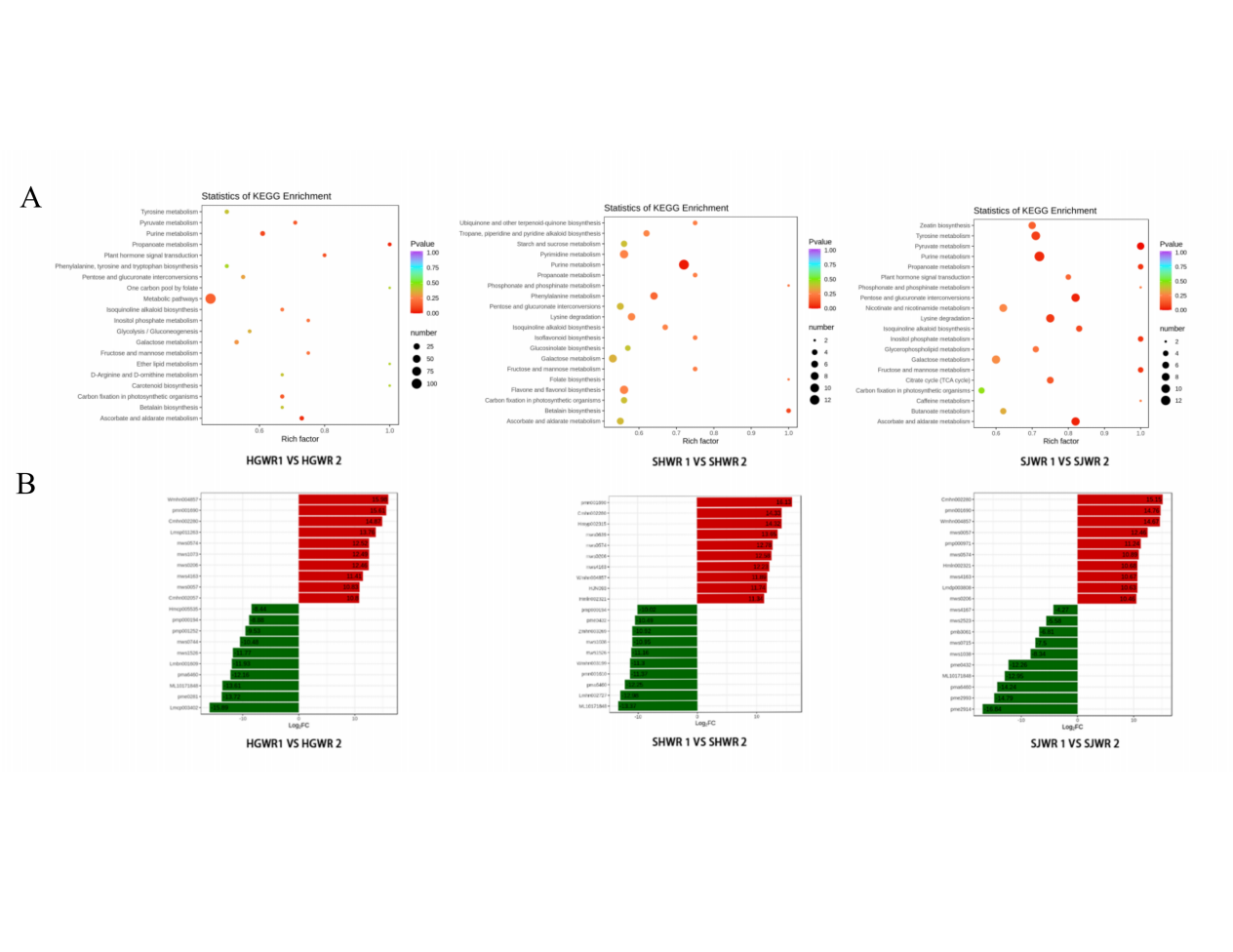


**Fig. S5.** **Enrichment analysis of metabolites and distribution patterns of the top 10 differential metabolites in three walnut cultivars.** (A) Differential metabolite enrichment analysis was performed using KEGG pathways. The horizontal axis displays the corresponding Rich factor for each pathway, while the vertical axis shows the pathway name. The significance of the enrichment is represented by the color of the point, with redder points indicating greater significance. The size of each point indicates the number of differential metabolites enriched in each pathway. (B) Differential Metabolite Bar Chart. The x-axis displays the log2FC of the metabolites exhibiting differential expression, and the y-axis displays the differentially expressed metabolites. Upregulated differentially expressed metabolites are depicted in red, while downregulated differentially expressed metabolites are depicted in green.


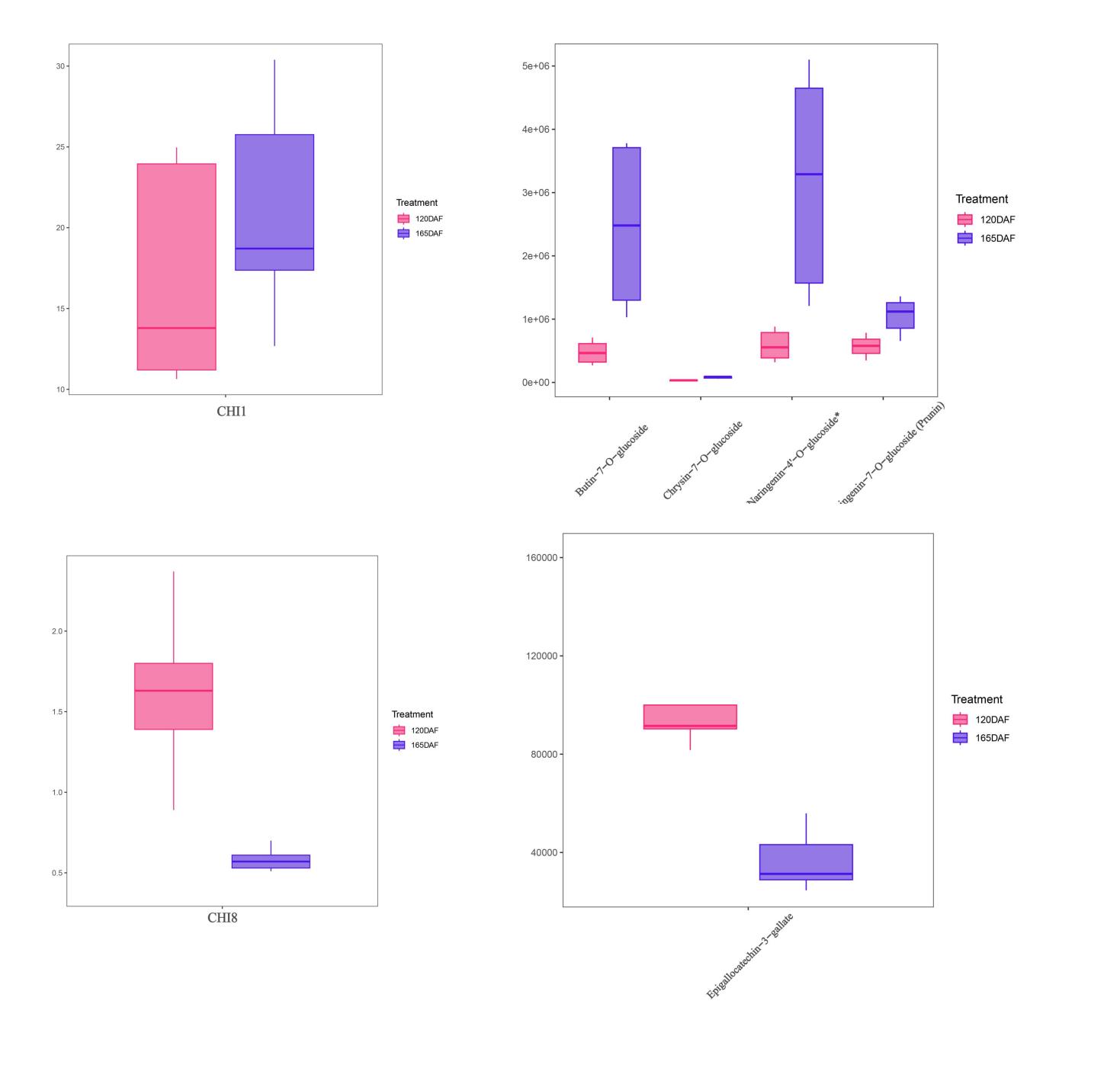


**Fig. S6 Differential expression of genes and metabolites at two walnut endopleura stages.**
